# Patient Stratification in Sepsis: Using Metabolomics to Detect Clinical Phenotypes, Sub-Phenotypes and Therapeutic Response

**DOI:** 10.3390/metabo12050376

**Published:** 2022-04-21

**Authors:** Humma Hussain, Kritchai Vutipongsatorn, Beatriz Jiménez, David B. Antcliffe

**Affiliations:** 1 Division of Anaesthetics, Pain Medicine and Intensive Care, Department of Surgery and Cancer, Faculty of Medicine, Imperial College London, London SW7 2AZ, UK; h.hussain@imperial.ac.uk (H.H.); kritchai.vutipongsatorn14@imperial.ac.uk (K.V.); 2 Section of Bioanalytical Chemistry, Division of Systems Medicine, Department of Metabolism, Digestion and Reproduction, Imperial College London, London SW7 2AZ, UK; b.jimenez@imperial.ac.uk; 3 National Phenome Centre, Department of Metabolism, Digestion and Reproduction, Imperial College London, London W12 0NN, UK

**Keywords:** sepsis, metabolomics, sub-phenotyping, organ dysfunction, patient stratification, NMR, LC-MS

## Abstract

Infections are common and need minimal treatment; however, occasionally, due to inappropriate immune response, they can develop into a life-threatening condition known as sepsis. Sepsis is a global concern with high morbidity and mortality. There has been little advancement in the treatment of sepsis, outside of antibiotics and supportive measures. Some of the difficulty in identifying novel therapies is the heterogeneity of the condition. Metabolic phenotyping has great potential for gaining understanding of this heterogeneity and how the metabolic fingerprints of patients with sepsis differ based on survival, organ dysfunction, disease severity, type of infection, treatment or causative organism. Moreover, metabolomics offers potential for patient stratification as metabolic profiles obtained from analytical platforms can reflect human individuality and phenotypic variation. This article reviews the most relevant metabolomic studies in sepsis and aims to provide an overview of the metabolic derangements in sepsis and how metabolic phenotyping has been used to identify sub-groups of patients with this condition. Finally, we consider the new avenues that metabolomics could open, exploring novel phenotypes and untangling the heterogeneity of sepsis, by looking at advances made in the field with other -omics technologies.

## 1. Introduction

Sepsis is a life-threatening condition [1] which results from a dysregulated immunological response to infection leading to organ dysfunction and often death [2], if not treated quickly and effectively [3]. Septic shock is the most severe form of sepsis, defined as the need for vasopressors to achieve a mean arterial blood pressure of 65 mmHg with a lactate greater than 2 mmol/L [2]. Globally, over 48.9 million people are affected by sepsis each year, with 11 million deaths annually [4]. The mortality associated with sepsis and septic shock is high and may vary between 22% and 76% depending on age, comorbidities, access to health care and regional provision of health care [5]. Sepsis is the main cause of mortality in intensive care units (ICUs) [6,7]. Treatment for sepsis is limited to antibiotics, fluid therapy and cardiovascular support and for decades no new therapies have become available for use in routine clinical practice [7], making sepsis highly challenging to manage. Some of the inability to find new therapies for this condition is due to its heterogeneity, for example caused by the range of causative organisms, sites of infection and variability in host response, with some therapies likely to benefit some but not all patients.

Pathogenesis of sepsis is complex [8] but we are gaining some understanding of how it results in organ dysfunction at a cellular, molecular and organ level [9]. Several studies have illustrated a complex link between pathogen and host that together add to the heterogeneous manifestations of sepsis [10]. A number of mediators, for example cytokines, pathogen-associated molecular patterns and endogenous damage-associated molecular patterns, play a crucial role in the pathology of sepsis and several complex pathways are involved in this disease process leading to multiorgan failure. However, further pathological understanding is required to untangle some of the heterogeneity, develop new treatments, stratify patients into those most likely to benefit from novel therapies and track response to therapy [1]. Currently, metabolomics offers potential for this, as metabolic profiles reflect human individuality and phenotypic variation [11,12].

Metabolomics, also known as metabonomics, metabolic profiling or metabolic phenotyping, has been broadly used to account for the global measurement of metabolites contained in biofluids and tissues with the intent of understanding how they change as a result of applied factors such as lifestyle, environmental stress, disease and drugs [13,14]. Metabolic profiling is non-selective, producing global measurements of metabolites, making this an effective method to investigate several biological processes simultaneously and discover new biomarkers that could have clinical utility [15]. Metabolomics differs from other -omics technologies such as proteomics, genomics and transcriptomics as it represents the molecular phenotype of an organism taking into account effects of the genome, proteome and the environment [16]. Metabolomic studies are conducted using analytical platforms such as nuclear magnetic resonance (NMR) spectroscopy and mass spectrometry (MS) coupled with a chromatographic or other separation techniques including liquid chromatography (LC) and ultraperformance liquid chromatography.

In this review we will address the ways that a metabolomic approach can aid in identifying clinical sepsis phenotypes such as survivors and non-survivors and identify new sub-phenotypes that could help develop future treatment strategies. Along with detailing the way that detection of metabolic disturbance could help in distinguishing sub-groups of patients, this review also focuses on the limitations and future directions of this approach which have not been previously discussed in the review recently published by Trongtrakul et al. [17].

## 2. Metabolomics to Identify Diagnostically Useful Clinical Phenotypes of Patients

### 2.1. Metabolomic Studies Aiming to Differentiate Sepsis from Other Conditions

Although mortality from sepsis remains high, it has been generally declining, mainly due to rapid recognition and early intervention. As such, there has been a great interest in understanding how metabolic phenotyping could be used to improve diagnostic accuracy and provide earlier detection and treatment of sepsis. Studies have aimed to separate patients with sepsis from healthy controls or those with sterile inflammation known as the systemic inflammatory response syndrome (SIRS) [18] using metabolomic techniques. The metabolic changes found in sepsis patients in relation to SIRS or healthy controls have been summarised in Table 1. Apart from Liang et al. [19], who analysed urine samples from a large cohort of participants (septic vs. healthy control, *n* = 2628), and Neugebauer et al. [20], who focused on serum of septic and SIRS patients (*n* = 406), studies comparing sepsis to healthy controls or SIRS are generally small with a range of 2 to 84 septic patients and 6 to 74 healthy controls or SIRS per study.

#### 2.1.1. Mitochondrial Dysfunction and Energy Metabolism

**Figure 1:**
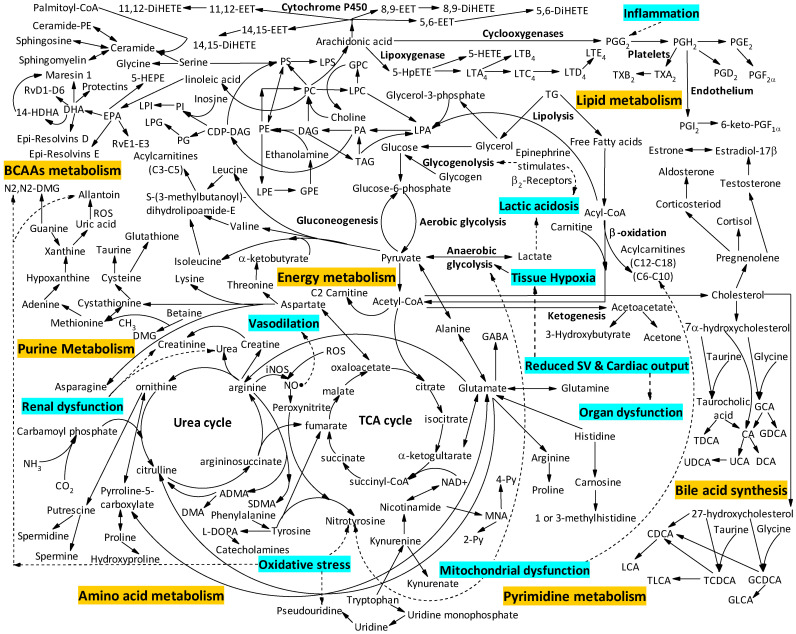
Main metabolic pathways involved in the pathology of sepsis identified using the Kyoto encyclopedia of genes and genomes (KEGG) [41]. Pathways highlighted in yellow correspond to metabolic derangements in sepsis and are related to: bile acid synthesis; energy metabolism; amino acid metabolism; purine and pyrimidine metabolism; lipid metabolism. The eight pathophysiological processes implicated in the metabolic response to sepsis are highlighted in turquoise: renal dysfunction; vasodilation; oxidative stress; mitochondrial dysfunction; lactic acidosis; tissue hypoxia; reduced stroke volume and cardiac output and organ dysfunction. Abbreviations: TG = triglyceride; PS = phosphatidylserine; PC = phosphatidylcholine; PE = phosphatidylethanolamine; PG = phosphatidylglycerol; PI = phosphatidylinositol; PA = phosphatidic acid; LPC = lysophosphatidycholine; LPE = lysophosphatidylethanolamine; LPG = lysophosphatidylglycerol; DAG = diacylglycerol; LPI = lysophospatidylinositol; TAG = triacylglycerol; DHA = docosahexaenoic acid; EPA = eicosapentaenoic acid; DiHETE = dihydroxyeicosatetraenoic acid; EET = epoxyeicosatrienoic acid; HpETE = hydroperoxy-eicosatetraenoic acid; HETE = hydroxyeicosatetraenoic acid; HDHA = hydroperoxy-docosahexaenoic caid; HEPE = hydroxypentaenoic acid; TX = thromboxanes; LT = leukotrienes; PGG_2_ = prostaglandin G_2_, PGH_2_ = prostaglandin H_2_; PGE_2_ = prostaglandin E_2_; PGI_2_ = prostaglandin I_2_; PGF_2α_ = prostaglandin F_2α_; keto-PGF_1α_ = keto-prostaglandin F_1α_; Rv = resolvin; CoA = coenzyme A; TCA = tricarboxylic acid; CA = cholic acid; DCA = deoxycholic acid; TDCA = taurodeoxycholic acid; GDCA = glycodeoxycholic acid; CDCA = chenodeoxycholic acid; LCA = lithocholic acid; GCDCA = glycochenodeoxycholic acid; TLCA = taurolitocholic acid; GLCA = glycolithocholic acid; DMG = dimethylglycine; DMA = dimethylamine; ADMA = asymmetric dimethylarginine; SDMA = symmetric dimethylarginine; GABA = γ-aminobutyric acid; NAD = nicotinamide adenine dinucleotide; 2-Py = N1-methyl-2-pyridone-5-carboxamide; 4-Py = N1-methyl-4-pyridone-5-carboxamide; L-DOPA = levodopa; NH_3_ = ammonia; CO_2_ = carbon dioxide; ROS = reactive oxygen species; CH_3_ = methyl; NO = nitric oxide; iNOS = inducible nitric oxide synthase; SV = stroke volume.

It is suggested that mitochondrial dysfunction in sepsis leads to the accumulation of glycolysis and tricarboxylic acid (TCA) cycle metabolites such as lactate, pyruvate and citric acid [16], and the pathways which lead to the accumulation of these metabolites are shown in Figure 1. Several studies in Table 1 consistently showed that sepsis patients had increased levels of glycolysis metabolites including lactate [21,22,23,24,25] and pyruvate [21,26] compared to healthy controls [21,22,23] or those with SIRS [24,25,26]. Lin et al. showed elevation of seven TCA metabolites as well as two TCA cycle derivatives including DL-gamma-methylketoglutaramate isomer and dimethyl fumarate in sepsis compared to healthy controls [21], which suggests upregulation of the TCA cycle in septic patients. Interestingly, Kauppi et al. and Jaurila et al. showed that the TCA cycle metabolite citric acid was significantly reduced in septic patients compared to healthy controls [26,27]. Additionally, the TCA cycle metabolite succinate was higher in sepsis patients than SIRS [25] which might indicate a shift in the general metabolism or mitochondrial dysfunction caused by the inhibition of succinate dehydrogenase (SDH) by itaconic acid or aromatic microbial metabolites (AMM) which act on the ubiquinone binding site of the respiratory chain [28]. Moreover, Beloborodova et al. also reported that patients with late-stage sepsis had higher concentrations of succinic acid and fumaric acid than patients with early-stage sepsis which could also be attributed to acidosis and tissue hypoxia seen in septic patients [28]. Other studies showed that short and medium chain acylcarnitines (including C3, C5, C6 (C4:1-DC), C8 and C10:1), which also accumulate in mitochondrial dysfunction due to altered fatty acid oxidation [29], were raised in septic patients compared to SIRS [20,30] suggesting enhanced incomplete β-oxidation as seen in Figure 1. The long-chain acylcarnitine C16:2(OH) was reduced in septic patients compared to SIRS [20], suggestive of reduced uptake of fatty acids into mitochondria as carnitines are involved in the transportation of long-chain fatty acids from cytoplasm [31]. In addition, studies focusing on adults and children also showed that patients with sepsis had increased levels of acetylcarnitine compared to SIRS [25,32] or healthy subjects [23], reflecting enhanced conversion of free carnitine and excess acetyl-CoA from metabolic stress into acetylcarnitine (Figure 1) via carnitine acetyl-transferase [33]. Moreover, sugars including glucose, mannose and lactitol dehydrate were reduced in septic patients compared to SIRS [25,32,34] whilst sucrose was elevated [25]. A hypoglycaemic state in septic patients might be induced by an increased conversion of glucose into pyruvate to meet increased energy requirements.

#### 2.1.2. Amino Acid Metabolism

Amino acids accumulate in the serum following breakdown of proteins and DNA in septic patients [16]. Several studies found higher levels of amino acids, such as glycine (a potent antioxidant [35]), leucine, isoleucine, glutamine, glutamic acid, cysteine, methionine, aspartic acid and lysine, in septic patients compared to healthy controls [19,21,26,27,36,37,38]. However, there are some inconsistencies in the branched-chain amino acids (BCAAs) with septic patients having been found to have both higher [21,37,38] and lower [25,26,39] levels compared to controls. These conflicting findings may be due to several factors including small sample sizes of the studies [21,25,26,37,38,39]. S-(3-methylbutanoyl)-dihydrolipoamide-E, a metabolite involved in the degradation of BCAAs (Figure 1) was elevated in sepsis patients compared to SIRS [34]. Moreover, the levels of isoleucine and leucine were reduced in septic infant [25] and paediatric populations [24] compared to controls, perhaps suggesting differences in metabolism between children and adults. Alterations in aromatic amino acids may relate to the increase in oxidative stress (Figure 1) with phenylalanine being higher in septic patients compared to healthy controls [21,37,38] those with SIRS [20,25,32] and in mechanically ventilated patients with pneumonia compared with those with brain injuries [40]. Similarly, Su et al. also reported higher levels of phenylalanine in severe sepsis compared to sepsis [38], suggesting that phenylalanine is not only a marker of sepsis but also of disease severity. Some conflicting results related to the aromatic amino acid tyrosine have been reported, with septic patients being seen to have both increased [37] and decreased [38] levels compared to healthy controls [37,38], perhaps as a result of differences in study design.

**Table 1:** Metabolomic studies focusing on the diagnosis of sepsis.

Study (Year)	Sample Type	Participants (Septic:Comparator)	Age Group	Comparator	Analytical Technique	Statistical Analysis Methods	Raised in Sepsis	Reduced in Sepsis
Li et al. [42] (2021)	Serum	84:59	Paediatric	Healthy control	HPLC-MS (targeted)	ANOVA, *t*-test, PCA, OPLS-DA, HCA, LR	D-mannose, d-quinovose, glycocholic acid, L-glutamate	PC (O-17:1/0:0), PI (20:4/18:1), PG (23:0/20:4), PE(P-17:0/0:0)
Mierzchala-Pasierb et al. [37] (2021)	Serum and urine	15:15	Adults	Healthy control	LC-MS (targeted)	MW-*U* test, *t*-test, Cox’s PHR, Spearman’s correlation, ROC analysis	Arginine, glycine, thioproline—in both. Histidine, a-aminoisobutyric acid, sarcosine aminoadipic acid, tyrosine, phenylalanine, leucine, lysine, isoleucine, ornithine, threonine, 4-hydroxyproline, glutamine 3-methyl-histidine, asparagine, aminopimelic acid—in serum. Citrulline—in urine	Histidine, aminoadipic acid, 3-methyl-histidine and aminopimelic acid—in urine. Dipeptide Gly-Pro—in serum
Jaurila et al. [27] (2020)	Serum	44:14	Adults	Healthy control	^1^H-NMR (targeted)	MW-*U* or *t*-test, FET, correlations	Glucose, glycine, creatinine, 3-hydroxybutyrate, glycoprotein acetyls mostly AGP	Citrate and histidine
Lin et al. [21] (2020)	Serum	31:23	Adults	Healthy control	GC–MS (targeted)	PLS-DA, *t*-test, ROC analysis, correlation analysis	Six FAs (including tetradecanoic acid [12-methyl-, methyl ester, (S)-], hexanoic acid, 2-methyloctadecanoic acid, palmitoleic acid, myristoleic acid, and 3-hydroxyoctanoic acid), amino acids (including leucine, glutamic acid, cysteine, methionine, phenylalanine, putrescine, and aspartic acid), six amino acid derivatives, twenty-seven organic acids (including lactic acid, adipic acid, and 3-hydroxypropionic acid), pyruvic acid and (NADP)-NADPH, two TCA cycle derivatives (including DL-gamma-methyl-ketoglutaramate isomer and dimethyl fumarate), seven TCA cycle metabolites	Two BCFAs (3-methyl-2-oxopentanoic acid, 4-methyl-2-oxopentanoic acid), ten saturated FAs (DPA, hexanoic acid, arachidic acid, palmitic acid, margaric acid, 10,13-dimethyltetradecanoic acid, nonadecanoic acid, pentadecanoic acid, propanedioic acid, methyl, ethyl ester, and stearic acid), nine unsaturated FAs (11,14-EDA, 11,14,17-ETA, adrenic acid, arachidonic acid, conjugated linoleic acid, bishomo-gamma-linolenic acid, linoleic acid, DHA, EPA), tryptophan, glutamine, serine, d-Proline, (N-methoxycarbonyl-, octyl ester), asparagine, 2 amino acid derivatives, citraconic acid, citramalic acid, DL-gamma-methylketoglutaramate isomer 1 and four TCA cycle metabolites
Sharma et al. [43] (2019)	Plasma	27:23	Adults	Healthy control	LC–MS/MS (targeted), ELISA tests	ANOVA, KWT	-	Total cholesterol, HDCH, LDCH, non-HDCH, Apo-A1, Apo-B100 andparaoxonase 1.
Sharma et al. [44] (2017)	Plasma	33:23	Adults	Healthy control	LC–MS/MS (targeted) and enzymatic colorimetry	Tukey’s HSD test, ANOVA	-	Total cholesterol, HDCH, LDCH, HDFC
Mecatti et al. [45] (2018)	Plasma and erythrocytes	20:20 (Sepsis and septic shock: healthy control)	Adults	Healthy control	LC-MS and GC-MS (targeted)	PCA, OPLS-DA	PCs (C15:0/18:2, C16:0/18:1) only in plasma. Total MUFAs, oleic acid (C18:1 n-9), PS (C18:0/22:1), PC (C16:0/20:4) only in erythrocytes. CL (1′ [18:0/18:2]/3′ [20:0/20:0]) and PC (C16:0/18:2 and C16:0/20:5) in both.	LPC (18:2/0:0), SM (d18:1/16:0), DHA (C22:6 n-3), PC (16:0/20:3) only in plasma. Total n-3 PUFAs, DPA (C22:5 n-3), PC (C15:0/18:2, C16:0/20:1) only in erythrocytes. Nine SMs (d18:1/20:1, d18:1/22:1 (d18:2/22:0), (d18:1/22:0)/(d16:1/24:0), (d18:0/22:0)/(d16:0/24:0), (d18:2/23:0), (d18:1/23:0), (d18:2/24:0)/(d18:1/24:1), (d18:1/24:0)/(d18:0/24:1), d18:1/17:0), 8 LPCs (15:0/0:0, 16:0/0:0, 18:3/0:0, 18:1/0:0, 18:0/0:0, 20:5/0:0, 20:4/0:0, 20:3/0:0), and PC (16:0/20:1) in both.
Szelig et al. [36] (2016)	Serum and urine	20:25 (Septic shock or severe sepsis:healthy control)	Adults	Healthy control	HPLC (targeted)	MW-*U* or *t*-test, Kruskal–Wallis and Spearman’s rho test	Serum meta-tyrosine on days 2 and 3. Urinary ortho-tyrosine on days 1 to 5, and urinary para-tyrosine on days 4 and 5.	Serum para-tyrosine on days 1 and 2. Urinary para-tyrosine on day 1.
Liang et al. [19] (2015)	Urine	1282:1346 (Septic shock: healthy control)	Adults	Healthy control	UPLC-MS (untargeted)	OPLS-DA, ROC analysis	Hippuric acid, 3-methyluridine, acetylcysteine	Kynurenic acid, glycine
Su et al. [38] (2015)	Serum	35:18	Adults	Healthy control	LC-MS/MS (targeted)	ANOVA, Χ^2^ and *t*-test, ROC curves, Pearson correlation.	Arginine, aspartic acid, homocitrulline, ethanolamine, glutamine, glutamic acid, phenylalanine, taurine, SAAs—on ICU admission	Cystathionine, EAAs, anserine BCAAs, BCAA/AAA ratio, asparagine, carnosine, citrulline, histidine, Isoleucine, isoleucine, valine, lysine, ornithine, phosphoethanolamine, proline, sarcosine, threonine, tryptophan, tyrosine—on ICU admission
Fanos et al. [22] (2014)	Urine	9:16	Neonates	Healthy control	^1^H-NMR and GC-MS (untargeted)	OPLS-DA	Lactate, glucose and maltose	2,3,4-trihydroxybutyric acid, rabitol 3,4-dihydroxybutanoic acid, ribonic acid, 3,4,5-trihydroxypentanoic acid, 2-ketogluconic acid, pseudouridine.
Mickiewicz et al. [23] (2013)	Serum	21:13—Infants 20:18—Toddlers 14:9—School Age	Infants, Toddlers, School Age	Healthy control	^1^H-NMR (targeted and untargeted)	PCA, PLS-DA, OPLS-DA, ROC analysis	2-Hydroxybutyrate, 2-hydroxyisovalerate, lactate in all three. Creatinine and 2-oxoisocaproate in infants and school age. Phenylalanine in school age and toddlers. 3-hydroxybutyrate, acetone, betaine, glucose and isobutyrate in toddlers. Arginine, carnitine, creatine, creatine phosphate, histidine, myo-inositol, O-acetylcarnitine in school age.	2-Aminobutyrate in infants and toddlers; acetate, adipate, threonine in infants. Glutamine in toddlers and citrate in school age.
Stringer et al. [46] (2011)	Plasma	13:6	Adults	Healthy control	^1^H-NMR (targeted)	Spearman’s correlation, *t*-test	Adenosine, Total Glutathione, PS	Sphingomyelin
Gaddnas et al. [47] (2009)	Serum	44:15 (Severe sepsis:healthy controls)	Adults	Healthy control	Radioimmunological assays	Χ^2^-test or FET, MW-*U* test, ROC analysis	Procollagen type III aminoterminal propeptides and crosslinked telopeptides of type I collagen.	-
Drobnik et al. [48] (2003)	Plasma	102:56	Adults	Healthy control	LC-MS/MS (targeted)	MW-*U* test, ROC analysis	Ceramides (C16:0, C18:0, C20:0, C22:1, C24:1 and total form), LPC (16:0, 18:0, 18:1, 18:2 and total form), LPC-PC ratio (16:0, 18:0, 18:1,18:2 and total form)	Ceramides (C23:0, C24:0)
Reisinger et al. [39] (2021)	Serum	52:25	Adults	ICU controls (without sepsis or bacteremia)	^1^H-NMR (untargeted) and Bruker IVDr lipoprotein analysis	LR, LMM, FET, Χ^2^ or MW-*U* test, PCA, PLS and OPLS-DA, correlations	VLPN, TG, V4 and V5TG, V2-V4FC, V4PL, L1TG, VLAB and ABA1	Valine, leucine, isoleucine, HDFC, H1-H3FC, HDCH, H1-H3CH, HDPL, H2-H4PL, TPA1, HDA1 and H1-H4A1
Grauslys et al. [24] (2020)	Serum	55:58	Paediatrics	SIRS	^1^H-NMR (targeted)	PCA, PLS-DA, *t*-test	3-hydroxtbutyrate, lactate, urea, valine, phenylalanine	2-hydroxyisobutyrate, acetate, acetone, leucine, pyruvate.
Antcliffe et al. [40] (2017)	Serum	15:26 (Pneumonia:brain injury)	Adults	SIRS (brain injury)	^1^H-NMR (targeted and untargeted)	PCA, OPLS-DA, CV-ANOVA, ROC analysis	Lipids, formate, phenylalanine, N and O-glycoproteins, unidentified metabolite (at 3.570–3.575 ppm). Lipoproteins (V5FC, L1-L4TG, L1PL, HDTG, H1and H2TG, L1 and L6AB).	Phospholipids (choline), glutamine and alanine. Lipoproteins (H3 and H4FC, L5 and L6FC, HDA1 and H4A1, TPA1 and TPA2, HDA2 and H4A2, HDCH, H3 and H4CH, L6CH, H4PL, L6PL).
**Neugebauer et al. [20]** (2016)	Serum	322:84 (Total) 123:42 (Test) 59:24 (Confirmation)	Adults	SIRS	LC-MS/MS (targeted)	MW-*U* test or *t*-test, HCA, LR, ROC analysis, KMSA	Serine, spermine, spermidine, aspartate, phenylalanine, total dimethylarginine, kynurenine, acetylornithine, acylcarnitine C6(C4:1-DC), PCaa (C32:0), SM (C16:1)	SM (C22:3, C20:2, C24:0, C26:1), SM-OH (C22:1, C24:1), LPCa (C24:0, C14:0), PCaa (C32:0, C32:2, C36:6, C40:4, C42:6), PCae (C44:4), acylcarnitine C16:2(OH)
Kauppi et al. [26] (2016)	Whole blood	65:49 (Bacteremic sepsis:SIRS)	Adults	SIRS	GC-TOF-MS (Untargeted)	H-MCR, OPLS-DA	Myristic acid, pyruvic acid	Isoleucine, norleucine, citric acid and a phosphocholine-like derivative
Mickiewicz et al. [32] (2015)	Serum and plasma	37:20 (Septic shock: SIRS)	Adults	SIRS	^1^H-NMR (targeted), cytokine and chemokine assay kits	PCA, OPLS-DA, ROC analysis	Proline, 3-hydroxybutyrate, isobutyrate, phenylalanine, myoinositol, 2-hyroxybutyrate, O-acetylcarnitine, urea, IP-10, HGF, IL-2Ra, IL-1Ra, IL-18	Valine, arginine, threonine, glutamate, glucose, methanol, propylene glycol, TNF-β, IL-1α, MCP-3
Su et al. [38] (2015)	Serum	35:14	Adults	SIRS	LC-MS/MS (targeted)	ANOVA, Χ^2^ test, *t*-test, Pearson correlation, ROC analysis	Homocitrulline, cystathionine, ethanolamine—at ICU admission	Anserine, phosphoethanolamine, lysine, δ-hydroxylysine, phosphoserine—at ICU admission
Mickiewicz et al. [25] (2014)	Serum	39:20 (Septic shock: SIRS)	Adults	SIRS	^1^H-NMR (targeted)	PCA, OPLS-DA, ROC analysis	Sucrose, lactate, myoinositol, proline, O-acetylcarnitine, isobutyrate, succinate, urea, creatinine, creatine, 2-hydroxyisovalerate, trimethylamine-N-oxide, 3-hydroxybutyrate, phenylalanine	Isoleucine, leucine, valine, lysine, glycine, serine, glutamine, alanine, threonine, glucose, mannose, glutamate, arginine, 2-aminobutyrate, methanol, 2-oxobutyrate, creatine phosphate
Su et al. [34] (2014)	Serum	35:15	Adults	SIRS	LC-MS/MS (targeted)	PCA, PLS and OPL-DA, MW-*U* test, ROC analysis	S-(3-methylbutanoyl)-dihydrolipoamide-E and N-nonanoyl glycine	Lactitol dehydrate and S-phenyl-D-cysteine
Mickiewicz et al. [23] (2013)	Serum	21:13—Infants 20:16—Toddlers 14:9—School Age	Infants, Toddlers, School Age	SIRS	^1^H-NMR (untargeted and targeted)	PCA, PLS-DA, OPLS-DA, ROC analysis	2-Hydroxybutyrate and glycerol in infants and school age. Glucose in infants and toddlers. Arginine in toddlers and school age. Citrate only in toddlers. Lactate, alanine, asparagine, creatine, creatine phosphate, creatinine 2-oxoisocaproate, ethanol, methanol, phenylalanine, taurine in school age.	Taurine in infants and toddlers. Adipate, alanine, glutamate, glycine, hopoxanthine, isoleucine, lactate, methionine, ornithine, serine, pyruvate, suberate, threonine in infants.
**Schmerler et al. [30]** (2012)	Plasma	69:74 (Total) 30:33 (Training) 39:41 (Test)	Adults	SIRS	LC-MS/MS (targeted)	MW-*U* or *t*-test, ROC analysis	Acylcarnitines (C3, C5, C6 (C4:1-DC), C8, C10:1). PCaa (C32:0, C34:1, C36:1), PCae (C34:1)	-
Su et al. [38] (2015)	Serum	12:23	Adults	Severe sepsis	LC-MS/MS (targeted)	ANOVA, Χ^2^ test, *t*-test, Pearson correlation, ROC analysis	Taurine (on days 1, 3, 5, 7, 10, and 14), cystine (on days 3, 7, 10, and 14), SAAs (on days 5, 10, and 14), whilst arginine, asparagine, aspartic acid, glutamic acid, leucine, serine, tryptophan, BCAAs, BCAA/AAA ratio—at certain time points	3-methyl-L-histidine, α-aminoadipic acid, α-amino-n-butyric acid, argininosuccinic acid, β-amino-isobutyric acid, carnosine, cystathionine, glutamine, phenylalanine, proline—at certain timepoints
Su et al. [34] (2014)	Serum	10:25	Adults	Severe sepsis	LC-MS/MS (targeted)	MW-*U*, PCA, PLS and OPL-DA, ROC analysis	Ne, Ne-dimethyllysine, glyceryl-phosphoryl-ethanolamine, 2-phenylacetamide, D-cysteine	-
Beloborodova et al. [28] (2019)	Serum	35:48 (Late: early-stage sepsis)	Adults	Early-stage sepsis	GC-MS	MW-*U*, Spearman’s correlation	Succinic acid, fumaric acid, p-HPhLA	-

Studies with validation cohorts are highlighted in bold. Abbreviations, LC: liquid chromatography; HPLC: high-performance liquid chromatography; GC: gas chromatography; MS: mass spectrometry; TOF-MS: time-of-flight mass spectrometry; MS/MS: tandem mass spectrometry; ^1^H-NMR: ^1^H-nuclear magnetic resonance; LR: logistic regression; LMM: linear mixed model; HCA: hierarchical clustering analysis; PCA: principal Component analysis; PLS-DA: partial least squares discriminant analysis; OPLS-DA: orthogonal projections to latent structures discriminant analysis; ANOVA: analysis of variance; CV-ANOVA: cross-validated ANOVA; MW-*U*: Mann-Whitney U; H-MCR: hierarchical multivariate curve resolution; KMSA: Kaplan-Meier survival analysis; ROC: receiver operating characteristic; Χ^2^: chi-square; FET: Fisher’s exact test; KWT: Kruskal-Wallis test; DHA: docosahexaenoic acid; DPA: docosapentaenoic acid; EPA: eicosapentaenoic acid; EDA: eicosadienoic acid; ETA: eicosatrienoic acid; NADP: nicotinamide adenine dinucleotide phosphate; EAAs: essential amino acids; AAAs: aromatic amino acids; BCAAs: branched-chain amino acids; BCFAs: branched-chain fatty acids; SAAs: sulphur containing amino acids; AGP: alpha-1-acid glycoprotein; Cer: ceramide, SM: sphingomyelin; PC: phosphatidylcholine; LPC: lysophosphatidylcholine; GPC: glycerophosphocholine; PS: phosphatidylserine; PI: phosphatidylinositol; PE: phosphatidylethanolamine; CL: cardiolipin; aa: diacyl; ae: acyl-alkyl; PUFA: polyunsaturated fatty acid; MUFA: monounsaturated fatty acid; HGF: hepatocyte growth factor; IL: interleukin; IP-10: interferon-inducible protein-10; Ra: receptor antagonist; p-HPhLA: p-(hydroxyphenyl)lactic acid; TG: triglyceride; HDL: high-density lipoprotein; LDL: low-density lipoprotein; VLDL: very-low-density lipoprotein; Apo-A1: apolipoprotein A1; Apo-A2: apolipoprotein-AII; Apo-B100: apolipoprotein B100; ABA1: apo-B100 to apo-A1 ratio; HDCH: HDL cholesterol; HDFC: HDL-free cholesterol; LDCH: LDL cholesterol; VLPN: VLDL particle number; H1-H4FC: HDL-free cholesterol subfractions 1 to 4; L5 and L6FC: LDL-free cholesterol subtractions 5 and 6; V2-V4FC: VLDL-free cholesterol subfractions 2 to 4; HDA1: HDL ApoA1; H1-H4A1: HDL Apo-A1 subfractions 1 to 4; TPA1: total plasma Apo-A1; TPA2: total plasma Apo-A2, HDA2: HDL Apo-A2; VLAB: VLDL Apo-B100; H4A2: HDL Apo-A2 subfraction 4; H1-H4CH: HDL cholesterol subfractions 1 to 4; L6CH: LDL cholesterol subfraction 6; HDPL: HDL phospholipid; H2-H4PL: HDL phospholipid subfractions 2 to 4; L6PL: LDL phospholipid subfraction 6; V5FC: VLDL-free cholesterol subfraction 5; L1-L4TG: LDL triglyceride subfractions 1 to 4; HDTG: HDL triglyceride; V4PL: VLDL phospholipid subfraction 4; H1 and H2 TG: HDL triglyceride subfractions 1 and 2; V4 and V5TG: VLDL triglyceride subfractions 4 and 5; L1 and L6AB: LDL Apo-B100 subfractions 1 and 6; TCA: tricarboxylic acid; C in sphingomyelin backbone represents the number of carbon atoms in the fatty acid side chain, whilst d in sphingomyelin backbone denotes 2 hydroxyl groups; SIRS: systemic inflammatory response syndrome.

Additionally, metabolomic studies in children revealed that phenylalanine levels were elevated in septic paediatric populations including toddlers and school age children compared to healthy controls [23] and those with SIRS [23,24]. Moreover, septic patients also had higher levels of creatinine, creatine, proline (involved in the synthesis of DNA, ornithine and polyamines via pyrrolline-5-carboxylate [35]), total dimethylarginine (a competitive inhibitor of nitric oxide (NO) [49]), kynurenine (a breakdown product of tryptophan [50]) and cystathionine (involved in synthesis of antioxidants [51]) compared to SIRS [20,25,32,38], whilst alanine (a substrate for glucose synthesis in the liver and energy substrate for leucocytes [35]), lysine (a regulator of NO synthesis [35]), 2-aminobutyrate, threonine (an inhibitor of apoptosis and stimulator of lymphocyte proliferation [35]), glutamine (a regulator of glutathione production [35]), glutamate and creatine phosphate were reduced compared to SIRS [25,32,40] and severe sepsis [38] (Figure 1).

#### 2.1.3. Urea Cycle

Arginine, an intermediate in the urea cycle is also involved in the synthesis of proteins, polyamines, creatine and NO [49,52] (Figure 1). In addition, it also takes part in several crucial processes including wound healing, cellular regeneration, protein turnover and immune function [52]. Adult septic patients have been reported to have both increased [37,38] and decreased [25,32,38] levels of the urea cycle metabolites arginine [25,32,37,38] and ornithine [37,38] compared to healthy controls [37,38], SIRS [25,32] and severe sepsis [38]. Argininosuccinic acid and citrulline were reduced [38], whilst aspartate, a precursor of the urea cycle (Figure 1), was elevated in septic patients compared to healthy controls [20,21,38] and severe sepsis [38]. Toddlers and school age children showed increased levels of arginine in septic populations compared to healthy controls or those with SIRS [23]. Urea, the end product of the urea cycle, was elevated in both septic adult [25,32] and paediatric [24] populations compared to SIRS [24,25,32] which might suggest renal dysfunction resulting in reduced elimination due to acute kidney injury, which is common in sepsis (Figure 1).

#### 2.1.4. Lipoproteins and Lipids

Levels of circulating lipoproteins, and lipids in general, decrease in critical care patients. In particular, the circulating levels of low-density lipoproteins (LDL) and high-density lipoproteins (HDL) have been observed to drop by up to 50% in septic patients [53,54]. High-density lipoproteins are described to have anti-inflammatory, anti-apoptotic and antioxidant effects, as they carry cholesterol back to the liver to be converted into bile salts and ultimately excreted from the body. All lipoprotein classes including HDL, LDL and very low-density lipoproteins (VLDL) can bind bacterial lipopolysaccharide (lps), but it is HDL that predominantly binds lps and lipoteichoic acid (LTA) [54]. For this reason, it has been suggested that HDL particles could have a therapeutic role in the treatment of sepsis [54]. Three metabolomic studies on adults reported that total cholesterol; non-HDL cholesterol; apolipoprotein (Apo)-A1, a major component of HDL [55] through which HDL binds to lps [56]; Apo-B100, a component of LDL, VLDL and intermediate-density lipoprotein (IDL) which is involved in the development of atherosclerosis [57]; HDL Apo-A1; HDL cholesterol; LDL cholesterol; and HDL-free cholesterol were reduced in sepsis patients compared to intensive care [39] and healthy controls [43,44], whilst VLDL-free cholesterol, triglycerides (TG) and VLDL triglyceride subfractions 4 and 5, VLDL Apo-B100 and Apo-B100 to Apo-A1 ratio were elevated [39]. These results suggest decreased neutralisation and clearance of bacterial toxins via the liver [58], increased TG production, reduced lipoprotein lipase activity and enhanced production or reduced clearance of VLDL [55,59]. Lipoproteins have also shown potential for differentiating patients with sepsis from pneumonia from other critically ill patients with brain injuries, with 27 lipoproteins differentiating the two conditions [40].

Interestingly, some of the most abundant phospholipids, such as phosphatidylcholines (PC), which are an integral part of the cell membrane [60], were found to be both increased [20,45] and decreased [20,30,42,45] in sepsis patients compared to healthy controls [42,45] and those with SIRS [20,30]. These phospholipids are important for the synthesis and stability of lipoproteins [60]. Only one metabolomic study in neonates showed that other phospholipids, including phosphatidylinositol (PI) 20:4/18:1, phosphatidylglycerol (PG) 23:0/20:4 and phosphatidylethanolamine (PE) P-17:0/0:0, were reduced in septic patients compared to healthy controls [42]. In addition, septic patients consistently had reduced levels of sphingomyelins (SMs) [45,46] and lysophosphatidylcholines (LPCs), a hydrolysis product of PCs [61], compared to healthy controls [45,46,48] and those with SIRS [20]. Cardiolipin (CL), a tetra-acylated diphosphatidylglycerol lipid situated in the inner mitochondrial membrane [62], is needed for mitochondrial respiration [63], and Neugebauer et al. reported that the ratio of unsaturated CL 1′ [18:0/18:2] to saturated CL 3′ [20:0/20:0] was elevated in septic patients compared to those with SIRS [45], indicating translocation of CL from the inner to outer mitochondrial membrane facilitating the release of proapoptotic factors [62]. Only one study looked into ceramides (Cer), the bioactive sphingolipids which are formed by the hydrolysis of sphingomyelins (SMs) [64] (Figure 1). These sphingolipids have a regulatory role in immune cell functions and activate apoptosis [64]. In this study, most of the ceramides (including C16:0, C18:0, C20:0, C22:1, and C24:1) accumulated in septic patients, with the exception of C23:0 and C24:0 which were reduced compared to healthy controls. Moreover, ratios of total Cer-to-SM, and some subtypes (C16:0, C18:0, C20:0, C22:0, C22:1, C23:0, C24:0 and C24:1) were also increased in septic patients [48]. Eicosanoids are bioactive lipids which are synthesised from C-20 polyunsaturated fatty acids (PUFAs) including arachidonic acid, homo-γ-linolenic acid and eicosapentaenoic acid (EPA) and they differ based on number of cis double bonds in the C20 carbon chain [65]. Eicosanoids take part in immunomodulation and other physiological processes [65,66]. Mecatti et al. reported that total n-3 polyunsaturated fatty acids, docosapentaenoic acid (DPA) and docosahexaenoic acid (DHA) were reduced [45] in sepsis patients compared to healthy controls. Similarly, Lin et al. showed that septic patients had decreased levels of the unsaturated fatty acids EPA and DHA compared to healthy controls [21], both of which are precursors to pro-resolving mediators, including resolvins, protectins and maresins, which are biologically active during tissue inflammation and organ injury [67]. In addition, total mono-unsaturated fatty acids and oleic acid (C18:1 n-9) were found to be elevated in sepsis patients compared to healthy controls [45], which might be due to increased lipolysis [68]. In addition, myristic acid, which is a monounsaturated fatty acid, was also elevated in septic patients compared to SIRS [26] and recently it has been reported that myristic acid could be a new potential candidate marker of sepsis, as it showed a high diagnostic sensitivity and specificity for identifying patients with bacteremia [69].

### 2.2. Metabolomic Studies Aiming to Identify Site of Infection and Causative Organism

Metabolic profiling also has the potential to help identify the anatomical location and causative organism of infection and understand differences in pathophysiology caused by these factors. Patients with severe urinary tract infections (UTIs) had increased levels of three LPCs (including LPCa (C16:0, C17:0, C18:0)) compared to patients with intra-abdominal infection. In addition, patients with UTIs had higher levels of acylcarnitine (C18:1); two biogenic amines, acetylornithine and taurine; six glycerophospholipids; and two sphingolipids than patients with blood stream infections (BSIs), whereas the glycerophospholipids PCaaC32:2 and LPCaC26:1 were reduced. Metabolic profiles of patients with intra-abdominal infections (IAIs) had elevated levels of acetylcarnitine and the biogenic amines compared to patients with BSI, whilst acylcarnitine (C18:1-OH) and the glycerophospholipid LPCaC26:1 were reduced [20].

The use of metabolic profiling may not only be helpful in determining the site of infection but in identifying the causative organism, thus allowing early identification of antibiotic-resistant pathogens and allowing more targeted antibiotic therapy. There is a large amount of metabolomic work aimed at improving identification of pathogens from laboratory samples and cultures, but this is outside the scope of this review. Here, we focus on studies that have aimed to understand the host metabolome in the context of different infecting organisms. One study revealed that three pathogens, *Staphylococcus aureus*, *Pseudomonas aeruginosa* and *Candida albicans* showed significant alterations in the levels of nine metabolites, including 1-oleoyl-L-alphalysophosphatidic acid, cholic acid, hypoxanthine, indoxyl sulfate, isovalerylglycine, histidine, PC(P-16:0/18:1), PI(16:0/18:3) and pregnenolone sulfate in sepsis survivors, non-survivors and the control group [42]. Sepsis survivors infected with *S. aureus* had higher levels of cholic acid, isovalerylglycine and histidine in comparison with those infected with *P. aeruginosa* and *C. albicans*; this could be attributed to immune responses or secondary to the pathogen metabolism [42]. Interestingly, another study revealed that there were no significant differences in the metabolic profiles of sepsis patients with *Streptococcus pneumoniae*, *Escherichia coli* and *Staphylococcus aureus* [70].

Despite the potential for metabolomic-derived biomarkers to aid in the rapid diagnosis of sepsis and identification of the causative organism, this approach has not yet made it into clinical practice due to lack of validation, low predictive capacity or limited reproducibility. Other problems which remain to be fully addressed include potential differences in metabolic profiles between children and adults. Although metabolic processes are broadly similar between adults and children, there appear to be some metabolites that have greater diagnostic potential in children than adults [22,23,24,42], implying that metabolic response may develop with age and suggesting that different diagnostic approaches may need to be taken in different age groups.

## 3. Metabolomics to Identify Prognostically Useful Clinical Phenotypes of Sepsis

### 3.1. Metabolomic Studies of Sepsis Survival

Several authors have used metabolomics to understand the metabolic perturbations caused by different severities of sepsis with the intent that this may aid prognostication and identify those most likely to benefit from novel treatments. Many of these studies have focussed on blood-based metabolic changes between survivors and non-survivors (Table 2). Although some of the findings of these studies support our current understanding and clinical features of more severe sepsis, for example that accumulation of bile acids associated with non-survival [71,72,73] is likely to relate to more frequent liver dysfunction in more severe sepsis, others allow novel insights and pathological understanding to be gained.

#### 3.1.1. Energy Metabolism

Lactate has been recognised as a clinical biomarker for the prognosis of sepsis for a long time and forms part of the current definition of septic shock [2]. Lactate production is increased as endogenous epinephrine stimulates glycolysis, leading to greater production of pyruvate than can be metabolised by the TCA cycle, leading to a move towards lactate production [74] (Figure 1). This is supported by several metabolomic studies which have found lactate [16,27,42,70,72,73,75] and pyruvate [16,70,75] to be elevated in sepsis non-survivors compared to survivors. In addition, an aromatic microbial metabolite, p-(hydroxyphenyl)-lactic acid (p-HPhLA) involved in the pathogenesis of septic shock [76], was also found to be increased in patients who die [70,72]. Many TCA cycle intermediates, including citrate, succinate, malate, fumarate and 2-ketoglutarate, are elevated in non-survivors [27,70,71,73,75], supporting the notion that the TCA cycle is upregulated (Figure 1) in an attempt to cope with the increased pyruvate production. Similarly, it is well recognised that stress-induced hyperglycaemia is a marker of poor prognosis in sepsis, although attempts to regulate glucose with insulin have had variable effect on outcome [77,78,79]. Metabolomic studies have once again confirmed elevated levels of carbohydrates, including glucose, fructose, sucrose, erythronate and mannitol, to be associated with non-survival [32,71,80,81] and accumulation of hippurate in non-survivors [73,80] could prevent the consumption of muscle glucose in patients with renal failure [82]. However, metabolomics has much more power than just confirming the relevance of clinical biomarkers. The accumulation of short and medium-chain acylcarnitines, including butyrylcarnitine, isobutyrylcarnitine, 2-methylbutyrylcarnitine, hexanoylcarnitine and decanoylcarnitine, as well as long-chain acylcarnitines, including dodecanoylcarnitine, palmitoylcarnitine and stearoylcarnitine, suggest mitochondrial and fatty acid oxidation dysfunction (Figure 1) in non-survivors [29,42,70,72,73,83,84]. Long-chain acylcarnitines have also been reported to inhibit pulmonary surfactant [29], thereby reducing lung function, providing a mechanism by which metabolite accumulation could lead to respiratory failure and worse sepsis severity. Betaine, a methyl donor involved in improving the impaired metabolism of sulphur-containing amino acids and oxidative stress [85], was found to be elevated in non-survivors [73], suggesting increased fatty acid oxidation and transport of hepatic lipids [85]. Increased catabolism of intracellular nicotinamide adenine dinucleotide (NAD^+^) leads to the formation of nicotinamide which is metabolized mainly into N1-methyl-2-pyridone-5-carboxamide (2-Py) [86] (Figure 1), which was found to accumulate in non-survivors compared to survivors [71], causing increased DNA damage and retention of catabolic products [87].

**Table 2:** Metabolomics studies of sepsis focusing on survival/death.

Study (Year)	Sample Type	Participants (Survivors:Non-Survivors)	Age Group	Analytical Technique	Statistical Analysis Methods	Raised in Non-Survivors	Reduced in Non-Survivors
Jones et al. [88] (2022) ^c^	Serum	113:39	Adults	UHPLC-MS (targeted)	PCA, PLSDA, FET MW-*U* test, MELR, Cox PHR, KMSA	14,15-dihydroxyeicosatrienoic acid (DHET)	-
Mierzchala-Pasierb et al. [37] (2021)	Serum and urine	11:4	Adults	UPLC-MS (targeted)	MW-*U* test, Cox PHR analysis	Serum 4-hydroxyproline and Glutamine	-
Li et al. [42] (2021) ^c^	Serum	74:10	Paediatric	HPLC-MS (targeted)	ANOVA, *t*-test, PCA, OPLS-DA, HCA, LR	Adenine, indolelactic acid, LPS (18:1/0:0), Ile-Tyr, kynurenine, glutamate, acetylcarnitine, tyrosine, tryptophan, palmitoylcarnitine, methionine, proline, acetylneuraminate and N2,N2-dimethylguanosine	PC (14:0/0:0,17:0/0:0, O-18:1/0:0), PI (18:0/22:5,18:0/22:6)
Reisinger et al. [39] (2021) ^c^	Serum	34:19 30:16 on days 3 and 7	Adults	^1^H-NMR (untargeted) and Bruker IVDr lipoprotein analysis	LR, LMM, FET, Χ^2^ or MW-*U* test, PCA, PLS and OPLS-DA correlations	-	BCAAs (valine, leucine, isoleucine)
Jaurila et al. [27] (2020)	Serum	33:11	Adults	^1^H-NMR (targeted)	MW-*U* or *t*-test, FET, correlations	Lactate and citrate	-
Khaliq et al. [89] (2020) ^c^	Plasma	12:8	Adults	LC-MS/MS (targeted) and industrial clinical chemistry system	PCA, mixed effects type-III ANOVA, Tukey HSD test, Random forests, linear SVMs	Aspartate aminotransferase (AST), alanine aminotransferase (ALT) and troponin T, putrescine, acylcarnitines (mostly short-chain acylcarnitines), amino acids (aspartate, tyrosine, phenylalanine, histidine) on days 0–3 or at any specific timepoint	HDCH, LDCH, 4 LPCs, 28 PCs, 11 SM-OH (C14:1, C16:1, C22:2, C23:0), SM (C16:0, C18:0, C18:1, C20:2, C22:3, C24:0, C26:0) on days 0–3 or at any specific timepoint
Wang et al. [16] (2020) ^c^	Plasma	134:54	Adults	LC-MS (targeted)	MW-*U* test, PLS-DA, ROC analysis	Isoleucine, alanine, acetylcarnitine, lactic acid pyruvic acid	LPG (22:0), and LPC (24:0)
Evans et al. [90] (2019) ^f^	Serum	7:4	Adults	LC-MS (untargeted)	MW-*U* test, Student’s *t*-test, generalised estimation equations	N-Methyl-phenylalanine, glucosamine, isoleucyl-proline/leucyl proline, histamine, adipoyl-L-carnitine, methoxytryptophol, fibrinopeptide A, N,N-dimethylguanosine, N-(3-acetamidopropyl)pyrrolidin-2-one, allysine	N-Acetyl-L-phenylalanine, phenylalanyl-tyrosine
**Chung et al. [91]** (2019) ^c^	Plasma	Derivation—69:21 Validation—96:24	Adults	UHPLC-MS (targeted)	Cox PHR, *t*-test, LR, ROC curves, KMSA	Acetylcarnitine (in both cohorts)	-
Huang et al. [92] (2019) ^e^	Plasma	63:30	Adults	UPLC-UV (targeted)	Χ^2^, MW-*U*, KWT and *t*-test, KMSA, Cox PHR, ROC	Leucine and phenylalanine	-
**Liu et al. [75]** (2019) ^b^	Serum	40:29 at 0 h 32:19 at 24 h	Adults	^1^H-NMR (untargeted and targeted)	PCA, OPLS-DA, *t*-test, ROC analysis	Lactate, pyruvate, alanine, glutamate, glutamine, lysine, 1-methylhistidine, tyrosine, phenylalanine, citrate at 0 h and 24 h. Methionine, fumarate, acetate, urea and 3-hydroxybutyrate at 0 h. Creatinine, 3-hydroxyisovalerate and lipids at 24 h	N-acetyl glycoproteins—0 h and 24 h
Cambiaghi et al. [93] (2018) ^c^	Plasma	9:8	Adults	LC-MS/MS (targeted)	Elastic net LR, LDA, PLS-DA	Day 7 to day 1 ratios of PCaa (C34:3, C36:3, C36:6, C42:1, C42:5), PCaeC30:1, SDMA, total dimethylarginine, proline, tyrosine	Day 7 to Day 1 ratios of LPC aC24:0, methionine, PCaa (C40:6, C42:6, C42:2), PCae (C30:2 and C42:5)
Winkler et al. [49] (2018) ^c^	Plasma	89:31	Adults	LC-MS/MS (targeted)	Χ^2^, MW-*U* test, KWT, KMSA	SDMA on days 1, 3 and 7, ADMA on days 1 and 3	-
Cirstea et al. [58] (2017) ^c,e^		186:14—Day 28 172:28—Day 90	Adults	Photometric analysis	KMSA, ROC analysis	-	HDCH
Dalli et al. [94] (2017) ^b^	Plasma	13:9	Adults	LC-MS/MS (targeted)	Wilcoxon paired signed rank test, FET, PLS-DA	PGF_2α_ (on days 0, 3 and 7), RvD5 (on days 3 and 7), RvE1, 17-HDHA, 18-HEPE, 15-HETE (on days 1 and 3), LTB_4_, RvE2,4S,14S-diHDHA and 5S,15S-diHETE (on day 7), 17R-PD1, 7-HDHA and 15-HEPE (on day 1), 17-epi-RvD1, 17-epi-PD1, 5-HETE and 5S,12S-diHETE (on day 3).	-
**Wang et al.** [95] (2017) ^c^	Plasma	CAPSOD—90:31 HAI–VAP—20:16	Adults	LC-MS/MS (targeted)	MW-*U* test, HCA, SVMs, ROC curves	Methylthioadenosine (MTA) in both cohorts	-
Sharma et al. [44] (2017) ^b^	Plasma	20:13—Day 1 14:9—Day7	Adults	LC-MS/MS (targeted) and enzymatic colorimetry	ANOVA, Tukey HSD test	No significant difference in lipoproteins.	
Liu et al. [73] (2016) ^b^	Serum	21:29	Adults	UPLC-MS (untargeted)	ANOVA, Tukey HSD test	Citrate, succinate, malate, α-ketoglutartae, amino acids (proline, BCAAAs, glutamine, glutamate, phenylalanine, betaine, creatine, creatinine, tyrosine), lactate, bile acids (GUDCA, GUDCS, GCDCA, GCA, UDCA), acyl carnitines (C6, C10, C12), indoxylactate, indoxysulfate, LPC 14:0	Ornithine, citrulline, argininosuccinate, acetylcarnitine, FFA (16:0,18:0), LPE (18:0,18:2,20:3,20:4), acylcarnitines (C16, C18)
Ferrario et al. [96] (2016) ^c,e^	Plasma	9:11 (90-day mortality) 11:9 (28-day mortality)	Adults	LC-MS/MS (targeted)	Unpaired Wilcoxon and paired Wilcoxon signed rank test, Multivariate Elastic Net regression analysis	Acetylcarnitine (on day 1) and kynurenine (on day 7)—based on 28-Day mortality.	PCs and LPCs species (on days 1 and 7)—based on 28-day and 90-day mortality. Six saturated long-chain LPC (aC16:0, aC18:0) and polyunsaturated very long-chain PC (aaC32:3, aaC34:4, aaC36:4, aeC40:1) at day 7—on both 28 and 90-day mortality.
Garcia-Simon et al. [80] (2015) ^d^	Urine	48:12	Adults	^1^H-NMR (targeted and untargeted)	ANOVA, PCA, PLS-DA, LR, ROC analysis	Ethanol, glucose, hippurate and an unknown metabolite (located at 1.40–1.45 ppm)—at 0 h and 24 h	Phenylalanine &arginine at 0 h and 24 h. Glutamine and methionine at 0 h
Su et al. [38] (2015) ^c^	Serum	20:15	Adults	LC-MS/MS (targeted)	ANOVA, Χ^2^ test, *t*-test, Pearson correlation, ROC analysis	α-aminoadipic acid, ethanolamine, cystathionine, and phenylalanine—at certain time points	Taurine (on days 10 and 14), BCAA/AAA ratio (on day 14), SAA (on days 7, 10, and 14) whilst arginine, glutamic acid, serine, and tryptophan at certain timepoints
Lee et al. [97] (2015) ^c^	Serum	65:52	Adults	Commercial kits with automated analysers	LMM, MW-*U* test or Student’s *t*-test, Cox PHR, ROC analysis	-	Cholesterol, TG, HDL, LDL, and Apo A-I—On days 0, 1, 3 and 7
Mickiewicz et al. [32] (2015)	Serum and plasma	8:8	Adults	^1^H-NMR (targeted) and cytokine and chemokine kits	PCA, OPLS-DA, ROC analysis	2-hydroxyisovalerate, fructose, IL-8, IL-9 and growth-regulated oncogene alpha (GRO-α).	Tumour necrosis factor (TNF)-β, beta-nerve growth factor (β-NGF) and dimethylamine
Kamisoglu et al. [84] (2015) ^c^	Plasma	90:31	Adults	LC-Q-orbitrap-MS and DSQ GC-MS (untargeted)	Welch’s *t*-test, Kolmogorov–Smirnov test	2-methylbutyroylcarnitine tiglylcarnitine, acetylcarnitine, hexanoylcarnitine octanoylcarnitine, propionylcarnitine, butyrylcarnitine, decanoylcarnitie, cis-4-decenoyl carnitine at 0 h and 24 h whilst deoxycarnitine only at 24 h.	1-eicosatrienoyl-GPC (20:3), 1-palmitoleoyl-GPC (16:1), 2-palmitoyl GPC (16:0) at 0 h and 24 h. 1-palmitoyl-GPC (16:0) 1-stearoyl-GPC (18:0), 1-oleoyl-GPC (18:1), 1-linoleoyl-GPC (18:2), 1-arachidonyl-GPC (20:4) at 24 h.
Mickiewicz et al. [25] (2014)	Serum	4:4	Adults	^1^H-NMR (targeted)	PCA, OPLS-DA, ROC analysis	20 metabolites were significant in differentiating survivors from non-survivors (results not reported)	-
**Rogers et al. [72]** (2014) ^c^	Plasma	RoCI—60:30 CAPSOD—115:34	Adults	GC-MS and LC-MS (targeted)	LR, Bayesian networks	Kynurenine lactate, p-HPhLA, ornithine, 3-hydroxyisovalerate, 2-hydroxyisovalerate, N-acetylalanine, sucrose, N-acetylserine, xanthine, allantoin, N2,N2-dimethylguanosine, 1-methylimidazoleacetate, glycocholate, GCDCA, TCDCA, taurocholate, cortisol, carnitines (C3, C4, C5, C5-OH, C5:1 and C6), γ-glutamylphenyl-alanine, γ-glutamyl-tyrosine—in both cohorts.	1-arachidonoyl-GPC (20:4), 1-arachidonoyl-GPE (20:4),), 1-palmitoyl-GPC (16:0), 2-palmitoyl-GPC (16:0), 1-linoleoyl-GPC (18:2), 1-stearoyl-GPC (18:0))—in both cohorts
Su et al. [34] (2014) ^a^	Serum	26:9	Adults	LC-MS/MS (targeted)	MW-*U*, ROC analysis, PCA, PLS and OPL-DA	S-succinyl glutathione, GPC, PG (22:2(13Z,16Z)/0:0), S-(3-methylbutanoyl)dihydrolipoamide-E)	-
Mickiewicz et al. [23] (2013)	Serum	10:10—Model 1 13:10—Model 2	Infants, Toddlers, School Age	^1^H-NMR (untargeted and targeted)	PCA, PLS-DA, OPLS-DA, ROC analysis	Eleven metabolites from model 1 and eighteen metabolites from model 2 were significant in separating survivors from non-survivors. (Metabolites not reported)	-
**Langley et al. [70]** (2013) ^c^	Plasma	Derivation set—90:31 CAPSOD—34:18 RoCI—36:25	Adults	UPLC-MS/MS (Targeted and untargeted) and GC-MS (untargeted)	ANOVA, LR, SVMs	Seventeen amino acid catabolites (lactate, p-HPhLA, 4-hydroxyproline, 3-methoxytyrosine), sixteen carnitine esters (Cis-4-decenoylcarnitine, 2-methylbutyroylcarnitine, butyroylcarnitine, hexanoylcarnitine), citrate, malate, pyruvate, dihydroxyacetone, phosphate, eleven nucleic acid catabolites, and four FFAs	Seven GPC and GPE esters, anabolic steroids, cortisone
**Seymour et al. [71]** (2013) ^e^	Plasma	15:15	Adults	UHPLC-MS/MS and GC-MS (untargeted)	Wilcoxon signed rank and *t*-test, random forests with supervised classification	Urea, cortisol, cortisone, fumarate, Kynurenate, 2-Py, pyridoxate, cofactors/vitamins, taurocholate, sulfated bile acids, sulfated hormones, N2, N2-dimethylguanosine, N1-methyladenosine, pseudouridine, allantoin, 10-hepatodecenoic acid, N6-carbamoylthreonyladenosine	GPEs and xenobiotics (paraxanthine and caffeine)
Gaddnas et al. [47] (2009) ^d^	Serum	33:11	Adults	Radioimmunological assays	Χ^2^ test, FET, MW-*U*, ROC	Procollagen type III aminoterminal propeptides and crosslinked telopeptides of type I collagen	-
Chien et al. [98] (2005) ^d^	Serum	44:19	Adults	Enzymatic and turbidimetric methods using kits	MW-*U*, FET, multivariate LR, KMSA	-	HDCH and Apo-A1 (on days 1 to 4)
Vermont et al. [99] (2005)	Serum	46:10	Paediatrics	Enzymatic colorimetric assay	Non-parametric test, FET	-	Total cholesterol
Drobnik et al. [48] (2003) ^d^	Plasma	63:39	Adults	LC-MS/MS (targeted)	MW-*U* test, ROC curves	Cer-SM ratios—on day 4 and day 11. Cer-SM to LPC-PC ratios—on day 1, 4 and 11	LPC-PC ratios—on day 4 and day 11
van Leeuwen et al. [53] (2003)	Plasma	10:7	Adults	DGU and enzymatic methods	ANOVA, *t*-test, MLRA	No significant differences in lipoproteins	-
Sprung et al. [100] (1991)	Plasma	10:5	Adults	Postcolumn IEC with ninhydrin detection	Spearman’s correlations, *t*-test	AAAs (tyrosine, phenylalanine), SAAs (taurine, methionine, and cysteine), ammonia and GABA	-
Roth et al. [81] (1982)	Plasma	7:7	Adults	Automatic amino acid analyser (Liquimat III)	Student’s *t*-test	Muscle valine and leucine and plasma levels of glucose, glucagon, phosphoserine, cysteine, valine, phenylalanine and 3-methylhistidine	Muscle glutamine, proline and lysine

Studies with validation cohorts are highlighted in bold. Abbreviations, UPLC-MS: ultra-performance liquid chromatography–mass spectrometry; UPLC-UV: ultra-performance liquid chromatography coupled to ultraviolet detector; UHPLC-MS: ultra-high performance liquid chromatography–mass spectrometry; GC-MS: gas chromatography coupled to time-of-flight mass spectrometry; DSQ GC-MS: dual-stage quadrupole gas chromatography-mass spectrometer; LC-MS/MS: liquid chromatography–tandem mass spectrometry; LC-Q-orbitrap-MS: liquid chromatography with quadrupole orbitrap mass spectrometry; IEC: ion-exchange chromatography, DGU: density gradient ultracentrifugation; PHR: proportional hazards regression; MW-*U*: Mann–Whitney *U*; LR: logistic regression; LMM: linear mixed model; MELR: mixed effect LR; MLRA: multiple-level regression analysis; HCA: hierarchical clustering analysis; LDA: linear discriminant analysis; PLS-DA: partial least squares discriminant analysis; PCA: principal component analysis; OPLS-DA: orthogonal projections to latent structures discriminant analysis; ANOVA: analysis of variance; HSD: honestly significant difference;; H-MCR: hierarchical multivariate curve resolution; KMSA: Kaplan-Meier survival analysis; ROC: receiver operating characteristic; Χ^2^: chi-square; FET: Fisher’s exact test; KWT: Kruskal-Wallis test; SVMs: support vector machines; AAAs: aromatic amino acids; BCAAs: branched-chain amino acids; aa: diacyl; ae: acyl-alkyl; PC: phosphatidylcholine; PG: phosphatidylglycerol LPC: lysophosphatidylcholine; PI: phosphatidyinositol; LPE: lysophosphatidyethanolamine; GPE: glycerophosphatidylethanolamine; GPC: glycerophopholcholine; Cer: ceramide, SM: sphingomyelin; Rv: resolvins; PGF2_α_: prostaglandin F2_α_; LT: leukotrienes; PD1: protectin D1; p-HPhLA: p-(hydroxyphenyl)-lactic acid; 2-Py = N1-methyl-2-pyridone-5-carboxamide; TCDCA: taurochenodeoxycholate; GCDCA: glycochenodeoxycholate; FAs: fatty acids; FFAs: free fatty acids; TG: triglyceride; HDL: high-density lipoprotein; LDL: low-density lipoprotein; HDCH: HDL cholesterol; LDCH: LDL cholesterol; Apo-A1: apolipoprotein-A1; TCA: tricarboxylic acid; SDMA: symmetric dimethylarginine; ADMA: asymmetric dimethylarginine; GABA: gamma-aminobutyric acid; HDHA: hydroxydocosahexaenoate; HEPE: hydroxyeicosapentaenoate; HETE: hydroxyeicosatetraenoate; CAPSOD: community acquired pneumonia and sepsis outcome diagnostics; RoCI: Brigham and Women’s Hospital Registry of Critical Illness. Symbols ^a–f^ represent censoring time point of mortality assessment; ^a^ 24-h mortality; ^b^ 7-day mortality; ^c^ 28-day mortality; ^d^ 30-day mortality; ^e^ 90-day mortality; ^f^ 1-year mortality.

#### 3.1.2. Amino Acid and Nucleotide Metabolism

Sepsis is characterised by patients entering a catabolic state resulting in the breakdown of proteins, carbohydrates and lipids [101] Many studies have found that protein catabolism is reflected in higher levels of BCAAs, such as leucine, isoleucine and valine, in the circulation of non-survivors [16,73,81,92]. However, these findings have not been universally confirmed and in some studies, BCAAs have been associated with better outcomes such as decreased ICU and 28-day mortality [39]. The levels of BCAAs and the ratio of BCAA to aromatic amino acids (BCAA/AAA) have been reported to be significantly lower in non-survivors in relation to survivors on day 14 [38,39], and higher levels of S-(3-methylbutanoyl)-dihydrolipoamide-E, a metabolite which plays a key role in the degradation of BCAAs (Figure 1), was elevated in non-survivors [34]. The importance of muscle catabolism in sepsis has been supported by the high levels of circulating glutamate and glutamine [42,73,75] along with the reduction in muscle glutamine in non-survivors [80]. It is interesting, however, that serum concentrations of glutamic acid were found to be significantly lower in non-survivors than survivors on day 7 following ICU admission [38], implying the dynamic nature of many of the metabolic changes associated with sepsis.

Changes in other amino acids, such as the aromatic amino acids, may relate to the increase in oxidative stress with tyrosine (a precursor for catecholamine production [35] (Figure 1)), 3-methoxytyrosine, and phenylalanine being consistently elevated in sepsis non-survivors [38,42,70,73,75,81,89,92,93,100], with the exception of one study which found that phenylalanine was reduced in this group [80]. Impaired function of the enzyme phenylalanine hydroxylase, which is involved in the synthesis of tyrosine, could lead to a change in the balance of phenylalanine and tyrosine [102]. Other amino acids, such as cysteine and taurine, which have anti-oxidant properties [35], and methionine, have been found to be both lower [38,93] and higher in non-survivors [42,75,81,100], perhaps reflecting differences in experimental design, analytical platforms and timing of sampling between studies. Cystathionine, an intermediate in the synthesis of the antioxidant amino acids cysteine and taurine (Figure 1), and α-aminoadipic acid, a marker of carbonyl oxidation in proteins [51], were found to be significantly elevated at ICU admission and on days 10 and 14 in non-survivors [38]. Non-survivors had elevated levels of indoxysulfate [73], a metabolite of tryptophan which causes oxidative stress by increasing the synthesis of reactive oxygen species, enhancing the activity of NAD(P)H oxidase, and reducing the levels of glutathione in the endothelial cells [103]. In addition, kynurenine, a breakdown product of tryptophan [50] (Figure 1), is an endothelium-derived relaxing factor [104] which was found to be accumulated in non-survivors [42,72,96] due to the release of cytokines [50] which are key mediators in the pathogenesis of sepsis and organ dysfunction [104].

Other metabolites associated with oxidative stress found to be elevated in non-survivors include those involved in pyrimidine and purine metabolism, including allantoin, pseudouridine, xanthine, N2,N2-dimethylguanosine, N6-carbamoylthreonyladenosine and N1-methyladenosine [42,70,71,72] (Figure 1). In addition, competitive inhibitors of NO including symmetric dimethylarginine (SDMA) and total dimethylarginine (DMA) were upregulated in patients who died indicative of oxidative stress [49,93] (Figure 1). NO is implicated in sepsis-induced vasodilation and is an important mediator in the immune response to infection [49]. Lysine has a regulatory role in NO synthesis [35] and has been found to be elevated in non-survivors [75] whilst muscle lysine was reduced [81], providing evidence for the importance of dysregulation of this pathway in the most severe forms of sepsis. Proline, a secondary amino acid that accumulates in response to stress and is a substrate for superoxide radical production [105], was found to be elevated in non-survivors [42,73,93] in all but one study [81], supporting the importance of oxidative damage in mortality from sepsis. A post-translational metabolite of proline, 4-hydroxyproline, which is important for collagen synthesis, structure and strength [106], was also found to be elevated in non-survivors [37,70].

Serine, which plays a role in the synthesis of glucose in the liver and kidneys; glycine; ceramides; and phosphatidylserine [35] (Figure 1) were all found to be significantly lower in non-survivors than survivors on days 7, 10 and 14 [38]. These metabolites have been implicated in the inhibition of apoptosis and stimulation of lymphocyte proliferation [35]; reduced levels in non-survivors is commensurate with the immune dysfunction that occurs in the most severe forms of sepsis. In addition, phosphoserine, a post-translational metabolite of serine, was also elevated in non-survivors [81] as well as creatine and creatinine [73,75], likely a consequence of the kidney and liver dysfunction and increased protein breakdown known to occur in sepsis [107,108]. Histidine has anti-inflammatory and anti-oxidant roles [109] and has also been found to accumulate in patients who go on to die from sepsis [89], as are its post-translational metabolites including 1-methylhistidine and 3-methylhistidine [75,81] (Figure 1). This may result from increased proteolysis in skeletal muscle [110] in an attempt to control the high levels of oxidative stress.

#### 3.1.3. Urea Cycle

Metabolomic investigations have demonstrated the importance of the urea cycle in the severity of sepsis. The urea cycle metabolites ornithine, argininosuccinate, arginine and citrulline have been seen to be lower in those people who go on to die from sepsis compared to those who survive [73,80]. Taken together with the accumulation of ammonia [38,100] and aspartate [70,73,75], the precursor molecules of this cycle, this implies a downregulation of this metabolic pathway (Figure 1). Urea, a commonly measured clinical biomarker, is also known to be commonly elevated in patients with sepsis who die [71,75]; however, this is most likely not due to increased production but reduced elimination with acute kidney injury. Accumulation of toxic molecules such as ammonia and urea (Figure 1) may go part of the way to explaining the high incidence of delirium seen in septic patients.

#### 3.1.4. Lipoprotein and Lipid Metabolism

Derangements in fatty acid, lipid and lipoprotein metabolism have been seen to be a hallmark of sepsis. During sepsis, lipoprotein classes including HDL, LDL and VLDL bind to lps and are involved in its clearance via the liver [58]. As previously described, HDL is the lipoprotein which possesses the highest affinity for both the Gram-positive and Gram-negative bacterial toxins LTA [111] and lps [54,112] and binds them via apolipoprotein-(Apo)-A1, a major component of HDL [55]. HDLs have several different roles during the acute stress response, such as promoting clearance of bacterial toxins, reducing platelet aggregation, inhibiting endothelial cell activation and apoptosis [113] and supporting the corticosteroid stress response [56]. Reduced levels of HDL, LDL and Apo-A1 were consistently reported in non-survivors in comparison to survivors of sepsis [89,97,98], which could lead to worse outcomes through many of the mechanisms described above, but specifically, the inability to clear lps may lead to acute lung and kidney injury [114]. Cholesterol, an integral part of cell membranes, is crucial for the normal function of all cells [115] and is also reduced in non-survivors [89,97,99] in both paediatric [99] and adult populations [89,97], suggesting increased metabolism of cholesterol into several important compounds including bile acids and adrenal and gonadal steroid hormones [115]. Similarly, reduced levels of HDL cholesterol were found in non-survivors [58,98], and on day 1, levels of HDL cholesterol < 20 mg/dl and Apo-A1 < 100 mg/dl were associated with increased 30-day mortality, longer ICU stay and increased rates of hospital-acquired infections [98]. Likewise, Cirstea et al. also reported that low HDL cholesterol levels in clinically suspected sepsis were associated with increased 28-day and 90-day mortality and adverse hospital outcomes [58].

Phospholipids are an integral part of cell membranes which are needed for the synthesis and stability of lipoproteins [60]. The two most abundant phospholipids of biological cell membranes include PCs and PEs [60] and alterations in the molar ratio of PC to PE could also affect energy production in mitochondria [60]. LPCs are produced by the hydrolysis of PCs (Figure 1) and have immuno-modulatory roles, for example by regulating monocyte and macrophage function [61], and anti-haemostatic roles, such as the inhibition of platelet aggregation and stimulation of NO secretion in endothelial cells [116]. LPCs can have both anti- and pro-inflammatory effects [116]. The majority of PC and LPC species were significantly reduced in both 28-day and 90-day non-survivors [96], a finding supported by several other studies [16,42,89]. Only one study found a species of LPC, LPC 14:0, to be upregulated in non-survivors compared to survivors [73]. In addition, the LPC-PC ratio was significantly lower in non-survivors and was also a strong predictor of mortality in sepsis patients [48], indicating decreased catabolism of PC species.

Low levels of shingolipids, including SMs such as SM-OH (C14:1, C16:1, C22:2, C23:0) and SM (C16:0, C18:0, C18:1, C20:2, C22:3, C24:0, C26:0) [89], in non-survivors might indicate increased catabolism of SM by acidic sphingomyelinase enzymes during inflammation, causing accumulation of the SM metabolites, ceramides [117] (Figure 1) which are involved in the regulation of immune cell functions [48]. Ceramides (Cer) are structurally similar to lps and are also a ligand for the lps CD14 receptor [118]; hence, they may act as receptor agonists [119]. Increased catabolism of SM species was also seen in another study where the molar ratio of Cer-SM was higher in non-survivors and was reported to be a strong predictor of mortality [48]. In addition, ratios of Cer-SM to LPC-PC ratios were also higher in non-survivors [48].

Glycerophosphocholine (GPC) and the fatty acid 10-hepatodecenoic acid were higher in those who did not survive, whilst other phospholipids, including 2-oleolyl-glycero-phosphoethanolamine (GPE), 1-linoleoyl-GPE, 2-linoleoyl-GPE, 1-archhidonyl-GPE, 1-linoleoyl-GPC and species of GPC, were lower in non-survivors than survivors [70,71,72,73,84], suggesting a complex relationship between fatty acid metabolism and sepsis survivorship.

Phosphatidylglycerols (PGs) are anti-inflammatory agents and an integral part of lung surfactant [120]. The level of PG 22:2 (13Z, 16Z)/0:0) was found to be higher in non-survivors [34], whilst its catabolic product lysophosphatidylglycerol (LPG) 22:0 was lower in comparison with survivors [16]. LPGs cause activation of natural killer cell trafficking and modulate the activity of endothelial cells [116]. Phosphatidylinositols (PIs) play a key role in intracellular signalling and the levels of PI (18:0/22:5), and PI (18:0/22:6) were found to be reduced in the non-survivors [42]. Lysophosphotidylserine, a signalling phospholipid which enhances histamine release and eicosanoid synthesis, has been implicated as a pro-resolving lipid mediator in inflammation [121]. However, increased levels of lysophosphotidylserine (18:1/0:0) observed in patients who die indicate that this pro-resolving activity may be insufficient [42].

Lipid mediators play an important role in the acute inflammatory response and resolution of inflammation [122]. For example, oxylipins are lipid mediators which result from the enzymatic oxidation of poly-unsaturated fatty acids by cytochrome P450s, cyclooxygenases or lipoxygenases during inflammation or infection [123,124]. The oxylipin 14,15-dihydroxyeicosatrienoic acid (DiHETE) (Figure 1) was present in significantly higher concentrations in the serum of patients with septic shock who did not survive compared to survivors, and this oxylipin showed an association with more severe organ dysfunction, especially hepatic failure [88]. Epoxyeicosatrienoic acids, the precursors of the DiHETEs, have vasodilatory, anti-inflammatory and organ-specific roles [125,126,127,128] that may be important in the resolution of sepsis, so a shift in their metabolism towards DiHETEs may be detrimental. Eicosanoids, a subgroup of oxylipins including leukotrienes (LTs) and prostaglandins, also play an important role in immunomodulation and physiological processes [65,66] such as vasodilatation and inflammation [78]. Dalli et al. showed that inflammation-initiating mediators, including prostaglandin F_2α,_ an airway smooth muscle constrictor [129,130], and LTB_4,_ a leukocyte chemoattractant [131] (Figure 1), were significantly upregulated in patients who die [94]. In addition, pro-resolving mediators, including resolvins (Rv) derived from docosahexaenoic acid (RvD5 and 17-epi-RvD1) and eicosapentaenoic acid (RvE1 and RvE2) and protectins (17R-PD1 and 17-epi-PD1) were also elevated in non-survivors [94] (Figure 1). Resolvins play a role in regulating the expression of cyclooxygenase-2, an enzyme involved in the biosynthesis of prostaglandin-F_2α_ (PGF_2α_) [132] and causes subcellular localization of the enzyme 5-lipoxygenase, causing it to switch leukotriene production to lipoxin production [132].

#### 3.1.5. Steroid Metabolism

Corticosteroids are used as an adjunctive therapy in septic shock and have been found to consistently reduce the duration of shock and may improve survival [133,134,135], and metabonomic studies have provided insights into important alterations to the balance of steroid hormones in more severe forms of sepsis. Levels of the catabolic steroids cortisol (Figure 1) and cortisone and pregnenolone sulfate are elevated in non-survivors compared to survivors [70,71,72], whilst levels of anabolic steroids are reduced [70]. This imbalance, in part, accounts for the catabolic state seen in sepsis whilst also supporting the notion of relative adrenal insufficiency as a mechanism for the benefit of supplemental corticosteroids in septic shock.

### 3.2. Metabolomic Studies of Organ Dysfunction

Whilst identifying the metabolic differences between survivors and non-survivors can provide key insights into the pathological mechanisms underpinning more severe sepsis, it is of limited clinical utility unless a set of metabolites can be identified that either predicts mortality with a very high degree of certainty or provides insights that can lead to new or targeted therapeutic approaches. Perhaps more clinically useful would be the identification of a set of metabolites that could predict patients at risk of organ failure more rapidly than current clinical investigations allow, thus facilitating more timely provision of organ support or the development of therapies to prevent organ dysfunction.

Only few studies have focused on the identification of organ dysfunction in sepsis. Rogers et al. reported that the global profiling of plasma did not differentiate patients with early sepsis with acute respiratory distress syndrome (ARDS) from those without ARDS [136]. However, Dalli et al. reported differences in eicosanoids between patients with sepsis who developed ARDS and those who did not. Inflammation-initiating mediators, including PGF_2α_, Δ6-trans-LTB_4,_ 12S, Δ6-trans-LTB_4_ (on days 0, 3 and 7), PGE_2_ (on day 3), PGD_2_ (on days 0 and 3) and 5-hydroxyeicostetraenoic acid (5-HETE) (on day 0) were significantly elevated in sepsis patients with ARDS compared to those without [94]. Increased levels of these inflammatory mediators indicate upregulation of inflammatory prostaglandin and LTB_4_ pathways (Figure 1) which could lead to lung inflammation and alveolar damage. In addition, pro-resolving mediators, including RvD2 and RvD3 (on day 3); RvE3 and RvD6 (on days 3 and 7); 17R-RvD1 (on day 7); 10S,17S-diHDHA, an isomer of protectin D1 (on days 1, 3 and 7); 17R-PD1 (on Day 3); 5-HEPE, a metabolite of eicosapentaenoic acid; and 14-HDHA, an intermediate in the synthesis of maresin 1 [137] (on days 0 and 3) were also significantly increased in septic patients with ARDS compared to those without [94] (Figure 1). Another study investigating metabolic differences in mechanically ventilated septic patients found that proline, glycine, glutamine, methionine, acetylcarnitine and valerylcarnitine were associated with liberation from mechanical ventilation and vasopressors at 28 days [138].

Acute kidney injury (AKI) is a frequent complication of sepsis and is associated with higher mortality. Lin et al. reported that patients with sepsis-induced AKI had higher circulating phenylalanine than patients without and that this could also predict the risk of later kidney dysfunction [21]. In addition, patients with AKI showed a reduction in 2-coumaranone [21] which has been associated with low vascular tone and which has some structural similarities with hydrolysed homocysteine thiolactone [139], a risk factor for arteriosclerosis, stroke and cardiovascular disease [140]. Although promising, no metabolomic approaches to predict organ dysfunction have reached clinical practice.

## 4. Use of Metabolomics to Identify Response to Treatment

Another potential strength of metabolomics is its ability to rapidly identify patients who are more likely to respond to treatments, are at risk of complications or track response to therapy. The power of this approach was demonstrated by Stebbing et al. who showed that metabolic profiles of patients with breast cancer could be used to predict response to chemotherapy [141]. However, such an approach has rarely been used in sepsis, with only a few studies using metabolomics to track response to sepsis treatment [90,142,143]. One study examined if metabolic profiling could be used to differentiate patients who were responsive to standard treatment, classified as improvement in organ dysfunction, from those who were not [142]. Analysis of untargeted data revealed that metabolic profiles varied by time from the start of treatment with metabolites such as lactate, pyruvate and histidine denoting treatment non-responders at 48 h after the onset of treatment [142]. Non-responders showed a steep decline in myristic acid; oleic acid; glutamine; and several phospholipids, including eight PCaa (C36:0, C36:3, C36:6, C38:3, C38:6, C40:5, C40:6, C42:2), 2PCae (C38:0 and C38:3), six LPCa (C16:0, C16:1, C18:0, C18:1, C18:2, C20:3) and two SMs (C18:0, C24:0); and elevation of alanine, phenylalanine, methionine and histidine [142]. The second study used serum samples collected alongside a clinical trial [144] to investigate metabolic signatures associated with improvement in vasopressor requirement in response to L-carnitine supplementation [143]. This study revealed that carnitine responders in a low-ketone group (3-hydroxybutyrate < 153 µM determined from pre-treatment samples) had elevated levels of phenylalanine, tyrosine, lysine and methionine, whilst carnitine and acetylcarnitine were reduced in comparison to other groups (carnitine responders in a high-ketone group and placebo patients in both low- and high-ketone groups) after 48 h of treatment. These patients also showed reduced 1-year mortality in relation to other groups [143]. In addition, this study also investigated the metabolic changes between survivors and non-survivors over time in L-carnitine-treated patients [143]. Ketone bodies including 3-hydroxybutyrate and acetoacetate were significantly elevated in non-survivors at baseline and after 24 h of treatment compared to survivors. In addition, 3-hydroxyisovalerate and creatine were also significantly elevated in non-survivors after 24 h of treatment whilst betaine and valine were reduced after 24 and 48 h of treatment, respectively [143]. Elevated levels of carnitine and acetylcaritine were also observed in non-survivors after 24 and 48 h of treatment with respect to survivors [143]. Patients who did not survive showed a greater disruption in carnitine metabolism with the acetylcarnitine to carnitine ratio (AC:C) being significantly higher in non-survivors prior to treatment, which then fluctuated more with time [143]. In another study of L-carnitine supplementation, within those patients who received L-carnitine, non-survivors had elevated levels of fibrinopeptide A, a marker of coagulation [145] which also induces C-reactive protein expression in vascular smooth muscles [146]; glucosamine, an anti-inflammatory agent formed during wound healing and tissue injury and repair [147]; histamine, which enhances vascular permeability [148]; allysine, a derivative of lysine involved in the synthesis and cross linking of collagen and elastin [149]; and N-(3-acetamidopropyl)pyrrolidin-2-one, a product of spermidine catabolism [150] in comparison to survivors, whilst phenylalanyl-tyrosine, a dipeptide of tyrosine and phenylalanine which is involved in the production of catecholamines [35], and N-acetyl-L-phenylalanine were reduced [90].

Although these studies have the potential for significant clinical impact by directing treatments to the right patients, there are a significant number of challenges to this approach. For example, data from observational studies are limited by potential baseline differences between patients who do and do not receive treatments that may confound metabolic analysis. Ideally to understand the impact of treatment on metabolic profiles, suitable samples need to be collected alongside large randomised clinical trials, as demonstrated above, where baseline differences are eliminated. Other challenges exist in the need to conduct longitudinal multivariate analysis over multiple time points and in selecting the correct sampling intervals to investigate potentially rapidly changing pathways.

## 5. Use of Metabolomics to Understand Changes to the Microbiome in Sepsis

In sepsis, the microbiome is greatly disrupted with a reduced overall microbial diversity, loss of beneficial flora and an excessive growth of potentially pathogenic bacteria, including *Enterococcus* and *Staphylococcus*. Pathogenic microorganisms are involved in the development of sepsis as perturbations in the gut microbiota can affect inflammatory responses and enhance the permeability in the gut barrier allowing the translocation of infectious agents to systemic circulation and other distant organs [151]. Understanding perturbations to the gut microbiota might provide a therapeutic approach for the prevention and management of sepsis, for example through modulation of microbiota by administering probiotics, enhancing the growth of beneficial flora by dietary interventions and prebiotics or total recolonization of the gut with a faecal microbiota transplantation [152].

Metabolomic approaches have the potential to provide a window onto the alterations in the microbiome by measuring bacteria-derived metabolites. Some metabolites are common to humans and bacteria whilst others are specific to one of these two organisms. Understanding alterations in these metabolites in septic patients could illuminate changes to the host-microbial metabolism integration [153] and might help us in understanding the disease development, progression and prognosis. Microbial metabolites are potential candidates for study as they are biologically active [154] and are involved in immune system regulation, central nervous system metabolism and epigenetic control in the host organism [155]. Beloborodova et al. have previously reviewed microbial metabolites and their roles in detail and grouped them into six main classes: (1) amino acids; (2) polyols; (3) fatty acids; (4) hydroxy acids; (5) amines and nitrogen heterocycles; and (6) nitrogen-containing bases of nucleic acids, nucleosides [153].

Microbial metabolites including short-chain fatty acids (SCFAs), such as acetate, propionate and butyrate, are produced by key bacterial species (*Faecalibacterium* spp., *Prevotella* spp., *Blautia* spp. and *Ruminococcaceae* spp.) which are an important part of the microbiota of healthy people but which decrease in the bowel of critically ill patients [156]. SCFAs play an important role in gut integrity and systemic immunity [156]. Low levels of SCFA acetate were found in patients with sepsis compared to healthy controls [23] or patients with SIRS [24] in a paediatric population supporting the loss of some of these bacterial species; however, the microbiota composition needs to be validated by in-depth sequencing techniques [156].

Microbial biodegradation products such as aromatic microbial metabolites (AMM) (including phenyllactic acid (PhLA), p-hydroxy-phenylacetic acid (p-HPhAA) and p-HPhLA) are the most important microbial metabolites which are synthesised from tyrosine and phenylalanine by several bacterial species, including *Staphylococcus aureus*, *Klebsiella pneumonia*, *Acinetobacter baumannii*, *Pseudomonas aeruginosa* and *Escherichia coli* [28,76]. AMM have already been proposed as metabolites with utility for assessing bacterial load [28] and were present in significantly higher concentrations in patients with septic shock compared to healthy controls [154] and non-septic patients with pneumonia [76]. This reflects reduced tyrosine hydroxylase activity, which limits the catecholamine production; decreased NO synthesis [157], which plays a role in microcirculation disorders and the progression of hypoperfusion; inhibition of inducible nitric oxide synthase (iNOS) expression [76,158]; and inhibition of succinate dehydrogenase (SDH) causing mitochondrial dysfunction [28]. Additionally, AMM could also act as a marker of severity as shown by Beloborodova et al. where late-stage sepsis patients had 30 times higher sum concentration of AMM than healthy controls, whilst it was only 10 times higher in patients with early-stage sepsis, indicating a disease continuum from health through to late-stage sepsis. This has also been suggested by the strong association of AMM with the SOFA score in patients with late-stage sepsis [28]. Disease progression was also seen in non-survivors having higher p-HPhLA levels than survivors [70,72] which reflects increased bacterial load and inhibition of NAD-dependent mitochondrial respiration in patients who die [154]. These studies show the potential of metabolomics to rapidly identify disruption to the microbiome without the need for bacterial culture. A metabolite-guided microbiota-targeted approach could be used to develop novel therapeutic approaches and could provide a means to monitor the response to these treatments.

## 6. Use of Metabolomics to Identify Novel Sub-Phenotypes

Application of unsupervised clustering techniques to other types of omics data have been successful in identifying sub-phenotypes of patients with sepsis that have different prognoses [159,160,161,162] and may respond differently to treatments [163]. These novel sub-phenotypes also allow new pathological insights to be gained that could lead to new therapeutic strategies. This approach has rarely been applied in the field of metabolomics. Hierarchical clustering of plasma metabolic profiles has revealed three distinct metabolic clusters with metabolic differences between groups mainly driven by plasma lipids. Group 1, associated with high mortality and rates of septic shock, had reduced levels of lipids compared to group 3, the lowest mortality group. These lipids mainly corresponded to fatty acid metabolites, lysophospholipids and sphingolipids [136]. Although this represents an exciting development, the findings have yet to be validated and the clinical utility of the three-group model have to be ascertained.

## 7. Discussion

Metabolomics studies have shown promise in helping better understand known clinical phenotypes of sepsis, especially the metabolic derangements associated with patients who go on to die. However, a limited amount of work has been carried out using this approach to identify novel sub-phenotypes and track response to treatment, although the potential has been demonstrated.

Broadly the same metabolic pathways were disrupted when patients with sepsis were compared to health or non-septic inflammation as when the severest sepsis, such as those who go on to die, were compared to less severe sepsis, suggesting that in many instances there is a continuum of metabolic disturbance from normal metabolism in health through inflammation to the most disturbance in the severest sepsis. In sepsis, there is an enhanced metabolic and energetic failure which leads to an increased demand for energy, and those requirements are met by increased glycolysis activity as well as by mitochondrial respiration [164]. Metabolic pathways including glycolysis and the TCA cycle (Figure 1) were upregulated in sepsis patients compared to healthy individuals and SIRS, as seen by the accumulation of glycolysis metabolites, lactate and pyruvate, and TCA cycle metabolites and their derivatives. Further upregulation was observed in patients with late compared to early sepsis and in those patients who went on to die. Similarly, mitochondrial dysfunction was observed in sepsis patients compared to SIRS as well as in non-survivors compared to survivors, again indicating disease progression.

Increased energy demand in sepsis leads to protein and amino acid metabolism [164]. Accumulation of BCAAs in sepsis patients compared to healthy controls as well as in patients who die may reflect disease progression; however, these finding were not universally confirmed as some studies showed reduced levels of BCAAs. Oxidative stress in sepsis is associated with an accumulation of aromatic amino acids and total dimethylarginine compared to healthy controls and SIRS as well as in non-survivors. A similar continuum of these disease sates is seen in the catabolic state induced by sepsis which causes breakdown of lipids and carbohydrates [101], downregulation of lipoproteins and lipids and alteration in the metabolism of phosphatidylcholines, which are crucial for the synthesis and stability of lipoproteins [60].

The improved understanding of the metabolic effects of sepsis has great potential to improve the clinical stratification of patients. Given that the biggest decrease in sepsis mortality have been due to improved recognition and detection of the condition which has allowed earlier intervention, it is likely that the first impact metabolomics will have is by improving clinicians’ ability to differentiate patients with sepsis from other forms of inflammation allowing earlier instigation of antimicrobials in those with sepsis and avoidance of these drugs in those who do not need them. However, there remain a number of uncertainties which will need to be addressed to take this into the clinical arena, for example, what is the optimal panel of metabolites to detect sepsis and how are they best measured. Further advances are likely to come from prognostic enrichment by early identification of sub-groups of patients more at risk of death or organ failure, facilitating more intensive monitoring or earlier intervention. However, as with differentiating sepsis from other conditions, limitations of current studies have prevented the adoption of this approach into hospitals.

Despite many consistent findings between metabolomics studies in sepsis, there are a number of findings that still need to be reconciled. For example, N-acetylglycoproteins, mostly α-1-acid glycoprotein which is an acute-phase protein [165] involved in systemic inflammation, were found to be higher in sepsis patients than controls [27] yet were lower in non-survivors with respect to survivors [75], and citrate was reduced in septic patients compared to healthy people [27] whilst being significantly raised in non-survivors compared to survivors [27,70,73,75]. Such conflicting findings may reflect the complexity of the underlying biological disturbance or be a feature of the heterogeneity we are keen to understand. However, it is equally possible that such inconsistencies reflect limitation of the current studies. With a few notable exceptions [16,19,20,48,88], most of the studies are small and often lack a validation cohort. Notably, we identified eight metabolomic studies which validated their findings [20,30,70,71,72,75,91,95], but two of these studies used mouse models for validation [71,95]. Other differences between studies may be accounted for by experimental design including the choice of control patients, time point of sampling and criteria used to define sepsis. Differences in the biofluids analysed and the platform used to generate metabolic profiles may impact on the experimental capability to detect metabolic differences between groups depending on metabolite abundance and sensitivity of the analytical platform. In the studies described in this review, there was a great deal of variation in the choice of platform used to generate metabolic profiles, with most utilizing NMR or a form of mass spectrometry; however, only one study harnessed the complimentary power of both [22]. Metabolomics can learn from the approaches taken by other -omics sciences by using large patient populations with well described validation cohorts. Future metabolomics studies in sepsis should aim to identify novel sub-phenotypes that may not be apparent to treating clinicians; as such, groups are likely to provide a greater understanding of pathophysiology and could be used to target treatments. Other future avenues to which to apply metabonomic methods are in identifying the metabolic responses to treatments by performing biological sampling and analysis alongside well conducted clinical trials, and deeper investigation of temporal changes in metabolic profiles over time and how they differ based on treatment, change in clinical condition and recovery.

## 8. Conclusions

Metabonomic studies have provided an important understanding of sepsis pathophysiology, especially the importance of metabolites related to energy metabolism, protein breakdown and lipid metabolism in patients with the severest sepsis. Such understanding is an important step in identifying sub-phenotypes within sepsis in order to reduce heterogeneity and better target treatment. Future studies are likely to utilize the power of unsupervised clustering, trajectory analysis and early assessment of treatment response to identify patients who are most likely to benefit from specific therapeutic approaches.

## Data Availability

Not applicable.
